# Ecological status assessment of Skalenski Lakes (Bulgaria)

**DOI:** 10.1080/13102818.2014.901682

**Published:** 2014-06-04

**Authors:** Ivanka Teneva, Gana Gecheva, Svetoslav Cheshmedjiev, Plamen Stoyanov, Rumen Mladenov, Detelina Belkinova

**Affiliations:** ^a^ Department of Botany, Faculty of Biology, Plovdiv University “P. Hilendarski”, Plovdiv, Bulgaria

**Keywords:** phytoplankton, functional groups, cyanotoxins, macrophytes, ecological assessment, lakes, WFD

## Abstract

Over the past decade new ecological indices based on phytoplankton and macrophytes were developed as part of the tools for assessment of the ecological status of water bodies. This study demonstrates the applicability of two of them (Assemblage index /*Q*/ and Algae Group Index /AGI/) for evaluation of water bodies from a lake type L4 as well as their comparability. Assessment of the ecological status of two lake ecosystems was performed in order to ensure successful protection, enhancement and management of lowland and semi-mountain lakes in Bulgaria. Data on the aquatic flora from Golyamo Skalensko Lake and Malko Skalensko Lake over a period of two years were used to assess their ecological status. In addition, the toxic potential of the established dominant cyanoprokaryotic species was also evaluated. Phytoplankton- and macrophyte-based metrics resulted in complementary evaluation of temporary and long-term environmental conditions. Despite the hydraulic connection and proximity between the two lakes, Golyamo Skalensko Lake and Malko Skalensko Lake appear as completely different ecosystems, according to the phytoplankton structure (species composition, number of species, abundance, seasonal succession), macrophytes and ecological status.

## Introduction 

In the EU Water Framework Directive (WFD), phytoplankton and macrophytes are identified as two of the biological quality elements (BQEs) which are required to assess the ecological status of lakes.[1] According to WFD, phytoplankton assessment should include the following metrics: taxonomic composition, species abundance or biomass, and frequency and intensity of phytoplankton blooms.[2] In the past decade, new ecological indexes based on phytoplankton were developed in many European countries as part of the tools for assessment the ecological status of water bodies.[3]

Aquatic macrophytes have also been widely used as indicators in lakes over the past decades.[4] They react progressively to environmental alterations such as nutrient loadings, but slowly, unlike phytoplankton.[5] Thus, they can be used as long-term indicators. According to WFD, ecological assessment has to be based on type-specific reference conditions and on aquatic flora composition and abundance.

Eutrophication is the most widespread pressure in European lakes, together with hydromorphological pressure.[6] In this context, it is necessary to identify which metrics are least affected by natural and methodological variation, and thus best reflect the most widespread pressures affecting the lakes.

In many aquatic systems Cyanobacteria play a major role as primary producers and may have a significant influence on other organisms in the ecosystem.[2] Under certain conditions, such as grading eutrophication of water bodies, higher water temperature, duration of sunlight and low values of the total nitrogen to total phosphorus (TN/TP) ratio,[7] Cyanobacteria could cause intense blooms. Most of these blooms are concomitant with the release of a large amount of cyanotoxins. Depending on their concentration in the water pool, these natural toxins could cause acute intoxication, could exert chronic effects or accumulate and pass through the food chain. That is why, these toxins and the species which produce them are focus of active research worldwide.[8]

This study included Skalenski Lakes, located in the Eastern Stara Planina Mountain. They are unique aquatic ecosystems that have not been investigated previously. The anthropogenic impact in these lakes is very weak and there are practically no sources of pollution. Our aims were (1) to study the phytoplankton, macrophytes and toxic potential of the established blue–green algae in the lakes; (2) to test the applicability of the Assemblage index for ecological assessment of semi-mountain natural lakes; (3) to perform ecological assessment of the lakes by using either the phytoplankton or macrophytes as indicators and to compare the two evaluations.

## Materials and methods

### Study area

**Figure 1.**
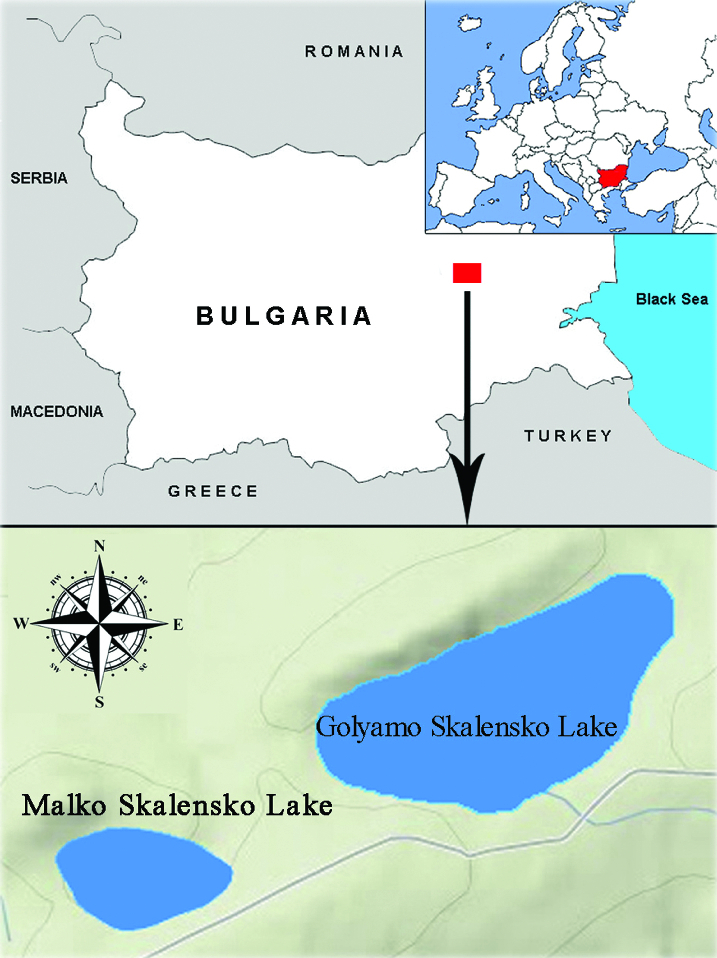
Location of the studied lakes.

Both Skalenski lakes fall within the catchment area of the river Luda Kamchia, Eastern Stara Planina Mountain (Figure 1). According to the adopted lake typology they belong to lowland or semi-mountain natural lakes and swamps in Pontic province (Lake Type L4).[9]

Golyamo Skalensko Lake (Reservoir Skala 1) is a natural semi-mountain lake, which is partially hydromorphologically modified and is rarely used for irrigation. Malko Skalensko Lake (Reservoir Skala 2) is highly modified and may be considered as an artificial water body. It was built in 1971 by pumping water from the river Luda Kamchia. Both lakes are near each other (500 m distance) and there is a hydraulic connection between them. The morphometric characteristics of Skalenski Lakes are given in Table 1.

**Table 1.** Morphometric, physical and chemical parameters of the studied lakes.

	Malko Skalensko Lake		Golyamo Skalensko Lake	
Parameters	Morphometric			
North latitude	42° 46′ 14.8″		42° 46′ 36.5″	
East longitude	26° 39′ 26.4″		26° 40′ 30.8″	
Altitude (m a.s.l.)	481		469	
Area (ha)	9.6		55.4	
Maximum depth (m)	8.5		11.0	
	Physical and chemical			
	2011	2012	2011	2012
*T* (°C)	26.3	21.1	25.4	19.6
DO (mg L^−1^)	7.98	7.32	7.82	7.62
OS (%)	96.1	93.1	93.0	84.4
рН	7.63	7.95	8.68	8.29
Cond (μS cm^−1^)	203	204	199	180
*T* (FAU)	6	13	15	27
N-NH_4_^+^ (mg L^−1^)	<0.01	<0.010	0.084	0.089
N-NO_2_^−^ (mg L^−1^)	<0.004	<0.004	<0.004	<0.004
N-NO_3_^−^ (mg L^−1^)	<0.04	<0.04	<0.04	<0.04
P-PO_4_ (mg L^−1^)	0.009	0.010	<0.003	0.006
TN (mg L^−1^)	<1.0	<1.0	<1.0	<1.0
TP (mg L^−1^)	0.017	0.019	0.028	0.031
COD (mg L^−1^)	7.9	8.3	10.5	9.7
BOD (mg L^−1^)	3.4	2.9	2.7	2.5

Note. T: temperature; DO: dissolved oxygen; OS: oxygen saturation; Cond: conductivity; T: turbidity; N-NH_4_^+^: ammonium nitrogen; N-NO_2_^−^: nitrite nitrogen; N-NO_3_^−^: nitrate nitrogen; P-PO_4_: phosphate phosphorus; TN: total nitrogen; TP: total phosphorus; COD: chemical oxygen demand; BOD: biochemical oxygen demand.

### Sampling procedure

Physicochemical parameters (temperature, pH, conductivity, transparency, dissolved oxygen and oxygen saturation) of lake water were measured *in situ*. Additionally, NH_4_-N, NO_2_-N, NO_3_-N, PO_4_-P, turbidity, total nitrogen and phosphorus, COD, and BOD_5_ were analysed immediately after sampling by using a NOVA 60 spectrophotometer (MERCK) following adopted standards: ammonium nitrogen (ISO 7150/1), nitrite and nitrate nitrogen (EN 26777 and ISO 7890-1), total nitrogen (EN ISO 11905-1), phosphate phosphorus (EN ISO 6878), total phosphorus (EN ISO 6878), turbidity (ISO 7027), biochemical oxygen demand (EN 1899-1,2) and chemical oxygen demand (ISO 15705).

Phytoplankton sampling was performed twice (July and September) in 2011 and 2012. Integrated samples were taken from the euphotic zone in the deepest part of the lake by a barometer type Ruttner (volume 2 dm^3^). The depth of the euphotic zone was defined as 2.7 times the Secchi depth.[10] Phytoplankton samples were preserved in formalin (4% final concentration). At the same time, non-preserved water samples were also collected and analysed for presence of cyanotoxins.

The taxonomic composition and abundance of aquatic macrophytes were recorded as biological metrics in the normative definitions of the ecological status classification in the WFD, Annex 1.2.[11] Sampling was carried out following the recommendations of Schaumburg et al.[12] The macrophyte surveys were carried out once during the main vegetation period (end of June until September). In each sampling site belt transects of 20–30 m width orthogonal to the shoreline and positioned within an ecologically homogenous section of the littoral were surveyed. The transect numbers were in correlation to the lake size. Species, their abundance and additional relevant parameters were recorded for the defined depth zones (0–1; 1–2; 2–4; and >4 m). The taxonomy of vascular plants followed Flora Europaea.[13,14] The abundance of each species was noted on a five-degree scale (1 = very rare, 2 = infrequent, 3 = common, 4 = frequent, 5 = abundant, predominant) according to Kohler.[15]

### Phytoplankton analysis

The taxonomic composition of the phytoplankton was determined by using an Amplival light microscope (400×). Live and preserved specimens were analysed. For taxonomic analyses additional live specimens were collected using a plankton net (35 μm). Guides and floras well established in the practice were used for identification of the taxa.[16,17,18,19,20,21,22]

Counting of the phytoplankton was performed by using an inverted microscope.[23] For numerous species, at least 100 specimens were counted.[24] The algal biovolume was calculated by using the formulas for geometric shapes.[25] Each translation between biovolume and fresh biomass was performed according to Wetzel and Likens.[26]

The assessment of the ecological status was made on the basis of five main and three additional metrics as follows. Main metrics: total biomass (mg L^−1^), Algae Group Index (AGI, Catálan Index), Assemblage index (*Q*), transparency according to Secchi (m) and Chlorophyll *a* (μg L^1^); additional metrics: percentage (%) of Cyanobacteria (towards total biovolume); bloom (intensity) and presence of bloom toxic species (*Microcystis*, *Aphanizomenon*, *Cylindrospermopsis* and others). The intensity of the phytoplankton bloom was assessed on the basis of total biovolume on a 5-degree scale.[27]

In calculating the percentage of Cyanobacteria, some species/genera for oligotrophic waters were excluded, focusing on toxic species and eutrophic indicators (according to WFD Intercalibration Technical Teport, Part 2-Lakes, Section 3-Phytoplankton biomass metrics Annexes).

AGI and Assemblage index (*Q*) were calculated as previously described.[28,29]

### Analyses for presence of cyanotoxins

#### ELISA analysis

Collected water samples were analysed by Ridascreen™ ELISA kit for detection of saxitoxins (R-Bopharm, Darmstadt, Germany), with a detection limit of 0.010 μg L^−1^; and by Microcystins ELISA kit (Abraxis LLC, Warminster, PA, USA) for detection of microcystins and nodularins, with a limit of detection of 0.10 μg L^−1^, according to the manufacturers’ instructions.

#### HPLC analysis

HPLC analysis was carried out using an ÄKTApurifier system (GE Healthcare Bio-Sciences AB, Uppsala, Sweden) and software UNICORN V5.11. An analytical column Discovery® C18 (5 mm × 4 mm, I.D. 5 μm) from Supelco (Bellafonte, PA, USA) was used. The mobile phase was a mixture of solution A (10 mmol L^−1^ ammonium acetate, pH 5.5) and solution B (10 mmol L^−1^ ammonium acetate:acetonitrile, 80:20, v/v) as follows: 0% B at 0 min, 100% B from 45 min to 65 min by using a linear gradient. The flow rate was 0.8 mL min^−1^ and ultraviolet detection was performed at 238 nm. All analyses were performed at room temperature. The column was equilibrated between the analyses with 8 mL of solution A. Each standard was assayed separately (nodularin 5 μg mL^−1^, MC-LR 5 μg mL^−1^, STX 40.5 pg mL^−1^, 200 μL injection volume), and then a mixture of all standards with the same concentrations (in 200 μL) was also analysed. The peaks of the samples were compared with those from the standards on the basis of the detection time of the peak.

#### **Macrophytes analysis**

Identified macrophyte species were allocated to the three different type-specific species groups: reference indicators, indifferent taxa, and degradation indicators (according to growth depth, some taxa are assigned to different groups). The Reference Index is an expression of the “plant quantity” ratio of type-specific sensitive taxa dominating at reference conditions compared to the “plant quantity” of insensitive taxa and is, therefore, a tool for estimating the deviation of observed macrophyte communities from reference communities. Calculated Reference Index [12,30] was transformed into Ecological Quality Ratio, where the value of “1” reflects the best possible ecological status.

#### **Classification system for assessment of the ecological status of lakes**

For evaluation of the ecological status of the lakes by using the phytoplankton and macrophyte metrics, we applied the classification system for a mesotrophic lake type in Bulgaria.[9] The scale for AGI (Catálan Index) is a modification after WFD intercalibration technical report, Part 2-Lakes, Section 3-Phytoplankton composition metrics (2008). Scales for chlorophyll-*a* and transparency are according to Cardoso et al.[31] The scale for Assemblage index (*Q*) follows Padisák et al.[29] The class boundaries for Reference Index were defined according to the normative definitions and interpretations of the WFD according to Gecheva et al.[30]

## Results and discussion

### Physicochemical parameters

The measured temperatures in the lakes (Table 1 **)** were typical for the summer. The pH values were slightly in the alkaline range, more pronounced in Golyamo Skalensko Lake. Nitrogen compounds were at the limit of the analytical method, which is typical for the epilimnion of stratified lakes during the summer According to the morphometric characteristics, Malko Skalensko Lake and Golyamo Skalensko Lake are small lakes of a medium depth, with pronounced summer stratification (Table 1). They are not subject to anthropogenic interference and should be expected to be in reference conditions.

### Phytoplankton

#### Taxonomic composition, species richness and biomass

**Figure 2.**
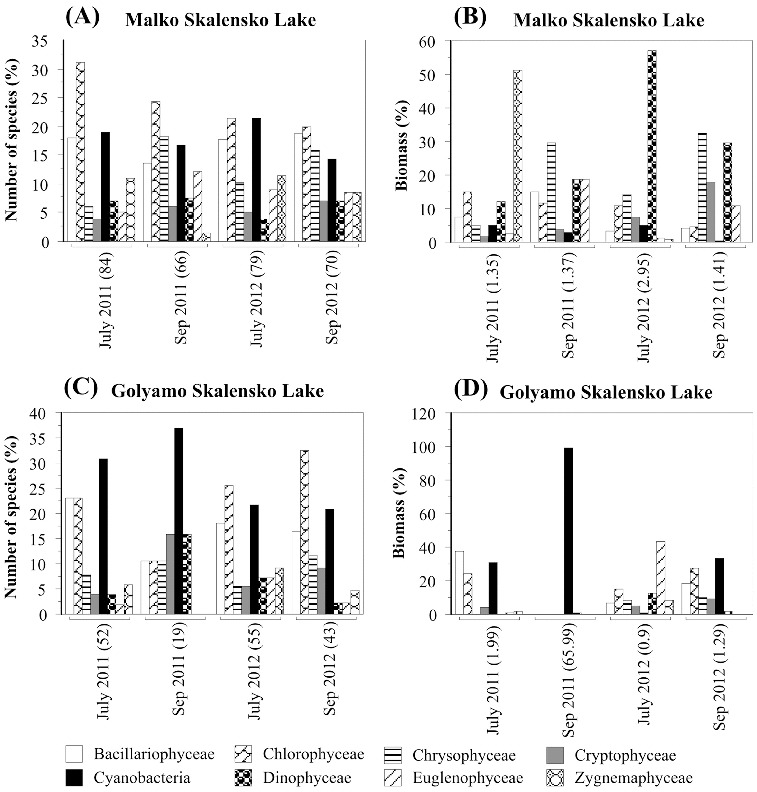
Relative abundance of the phytoplankton based on taxonomic groups. In parentheses: total species number and total biomass.

The summer phytoplankton of Malko Skalensko Lake featured high species richness, low biomass and stability over time (Figure 2(A)). Eighty-four species were identified in the samples collected in July 2011, and 66 species in those collected in September 2011. The species were distributed relatively evenly in eight taxonomic groups. The largest number of species in both months belonged to Chlorophyceae, 30.9% and 24.2%, respectively. In July, the second group in terms of species richness was Cyanobacteria (19.1%), followed by Bacillariophyceae (17.8%) and Zygnemaphyceae (10.7%). Towards the end of the summer, with the decrease in water temperature in September, the number of Chrysophyceae and Euglenophyceae species increased to 18.2% and 12.1%, respectively.

There were also a large number of species in the samples collected in 2012 (July and September), 79 and 70, respectively, belonging to eight taxonomic groups (Figure 2(A)). Analogously to 2011, in July 2012, Chlorophyceae (21.5%), Cyanobacteria (21.5%) and Bacillariophyceae (17.7%) predominated, while in September the number of Chrysophyceae (18.6%) was increased. Both in 2011 and 2012, a small number of eutrophic and toxic Cyanobacteria species (*Microcystis aeruginosa*, *Aphanizomenon flos-aquae*) were observed as single specimens only in the quality samples.

The total biomass of the phytoplankton in Malko Skalensko Lake ranged from 1.35 to 2.95 mg L^−l^ (Figure 2(B)). In July 2011, Zygnemaphyceae (51.3%) dominated in the biomass, followed by Chlorophyceae (14.8%) and Dinophyceae (12.2%). In September 2011, other taxonomic groups were of paramount importance for the biomass: Chrysophyceae (29.4%) and Euglenophyceae (18.9%). In July 2012, algal bloom (1st degree) of the dinoflagellate alga *Peridinium lomnickii* was observed and it represented 57.1% of the total biomass. In September, the bloom was decreased and, similarly to 2011, an increase in the relative abundance of Chrysophyceae (32.6%) and Dinophyceae (29.7%) was registered. It is noteworthy that, both in 2011 and in 2012, Cyanobacteria were represented by few eutrophic and toxic species. The relative biomass of Cyanobacteria in Malko Skalensko Lake was very low and ranged from 0.4% to 5.0% (Figure 2(B)), which corresponds to the absence of cyanotoxins in the analysed water samples.

The phytoplankton of Golyamo Skalensko Lake was characterized with lower species richness (from 19 to 55 species) and extraordinary dynamics in taxonomic composition and total biomass, without similarity between the months and years (Figure 2(C) and 2(D)). In 2011, blue–green algae clearly dominated in number of species: Cyanobacteria (30.8%), Chlorophyceae (23.1%) and Bacillariophyceae (23.1%). In September 2011, the number of Cyanobacteria species increased even more (36.9%). In 2012, the same taxonomic groups dominated, but the largest number of species was that of Chlorophyceae: 25.5% (July) and 32.6% (September), respectively. In both years, eutrophic and toxic species such as *Microcystis aeruginosa*, *Microcystis wesenbergii*, *Aphanizomenon flos-aquae* and *Anabaena spiroides* were also identified.

The total biomass of the phytoplankton in Golyamo Skalensko Lake for the two studied years varied in a very large range: from 0.9 to 65.99 mg L^−l^. In July 2011 and September 2012, the total biomass was dominated by Cyanobacteria, Chlorophyceae and Bacillariophyceae, with approximately the same percentage distribution. In September 2011, Cyanobacteria were the only dominant group, representing 98.9% of the total biomass. During this period, there was an intense bloom of *Anabaena spiroides* f. *crassa* (65.33 mg L^−l^, third degree), which resulted in extremely high levels of total biomass (65.99 mg L^−l^). In July 2012, unlike other months, the total biomass was dominated by Euglenophyceae (43.7%) and Chlorophyceae (14.6%).

#### Main species and functional groups (FGs) of phytoplankton

**Figure 3.**
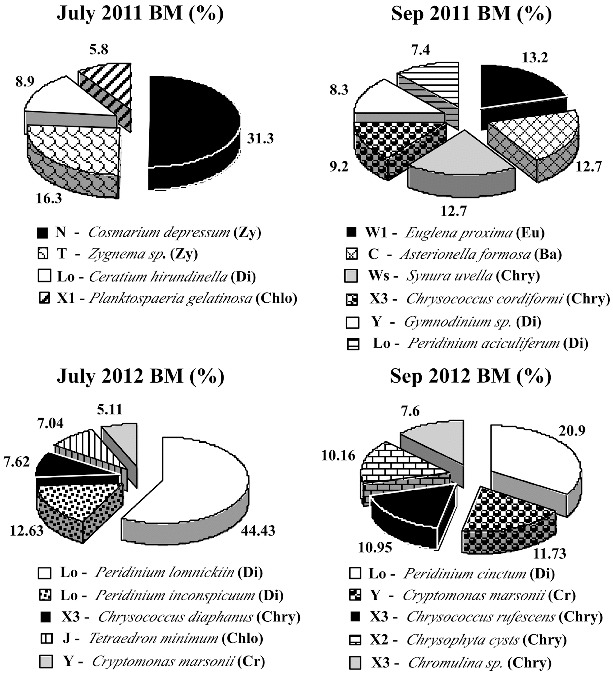
Relative biomass (Bm, %) of the main phytoplankton species (>5% of the total biomass) with their taxonomic and functional groups in Malko Skalensko Lake.

The main phytoplankton species in Malko Skalensko Lake belonged to 11 functional groups: **N**, **T**, **Lo**, **F**, **X3**, **X2**, **J**, **Y**, **W1**, **C** and **Ws** (Figure 3). Most of them inhabit pure oligo- and mesotrophic lakes. In July 2011, almost one-third of the total biomass (31.3%) in this lake was made up by functional group **N**, represented by the desmid alga *Cosmarium depressum*. Species from group **N** are known to grow during the summer in lakes of the temperate zone.[32] These species are tolerant to nutrient deficiency.

The second dominant species was *Zygnema* sp. (16.3%) from functional group **T**. This group inhabits swirling layers where light is a limiting factor.[33] *Ceratium hirundinella* (**Lo**) was also a dominant species (8.9%). Group **Lo** (mostly dinoflagellates) are distributed in a wide range of habitats, reflecting their ability to live in almost all types of ecosystems.[33] The motility of dinoflagellates allows efficient absorption of nutrients, and their mixotrophy and phagotrophy make them less dependent on the low levels of nitrogen and phosphorus.[34] One of the dominants in Malko Skalensko Lake in July 2011 was *Planktosphaeria gelatinosa* (5.8%) from group **F**, which is also tolerant to low nutrients.[32]

At the end of the summer, a change had taken place in the dominant functional groups in Malko Skalensko Lake (Figure 3). In September 2011, there were three key functional groups with equal amounts of biomass: **W1**, **Ws** and **C**. The predominance of **Ws** (*Synura uvella*, 12.7%) indicates presence of organic matter derived from decomposition of plant materials.[4,35] Chrysophycean algae from genus *Synura* grow in small lakes rich in organic matter from decomposition of plant materials. Often, the water is coloured by humic substances. Probably this is the reason for the reduced transparency in Malko Skalensko Lake (1.42 m) at the end of the summer and lowest environmental assessment of this indicator – moderate status. In this type of lakes, euglenoids are often subdominants of *Synura*.[35] This was also confirmed in Malko Skalensko Lake. In September, *Synura uvella* was associated with the euglenoid alga *Euglena proxima* (13.2%). The third dominant belonged to functional group **C**, which includes centric diatoms (*Asterionella formosa*, 12.7%). Species from group **C** are sensitive to silicon (Si) depletion and stratification.[32] Therefore, we believe that the prevalence of *Asterionella formosa* in the late summer in Malko Skalensko Lake is an indicator of increased mixing of the water and its enrichment with Si. Other groups with low relative abundance in September 2011 included **X3** (*Chrysococcus cordiformis*, 9.2%), **Y** (*Gymnodinium* sp., 8.3%) and **Lo** (*Peridinium aciculiferum*, 7.4%).

In July 2012, more than half of the phytoplanktonic biomass belonged to functional group **Lo**, represented by *Peridinium lomnickii* (44.43%) and *P. inconspicuum* (12.63%). The functional groups with less relative biomass were **X3**, **J** and **Y**. Group **X3** inhabits shallow oligotrophic environments [32] and was represented throughout the studied period by *Chrysococcus diaphanous* (7.62%), *Chrysococcus rufescens* (10.95%) and *Chromulina* sp. (7.6%). Representatives of functional group **Y** (mostly cryptomonads) inhabit a variety of aquatic systems, and they are sensitive only to the pressure of the zooplankton.[33] From this group, *Cryptomomas marsonii* was identified in the samples from both months (July 5.11% and September 11.73%). Functional group **J** (*Tetraedron minimum*), which is distributed generally in shallow, enriched lakes,[32] was found only in July 2012 in Malko Skalensko Lake (7.04%). Its co-existence with *Peridinium lomnikii* (which caused a first-degree bloom) is indication for a slight increase of the trophic level and good status in most phytoplanktonic indicators. At the end of the summer, in September, another dominant group was **X2**, represented by *Chrysophyceae cysts* (10.16%).

**Figure 4.**
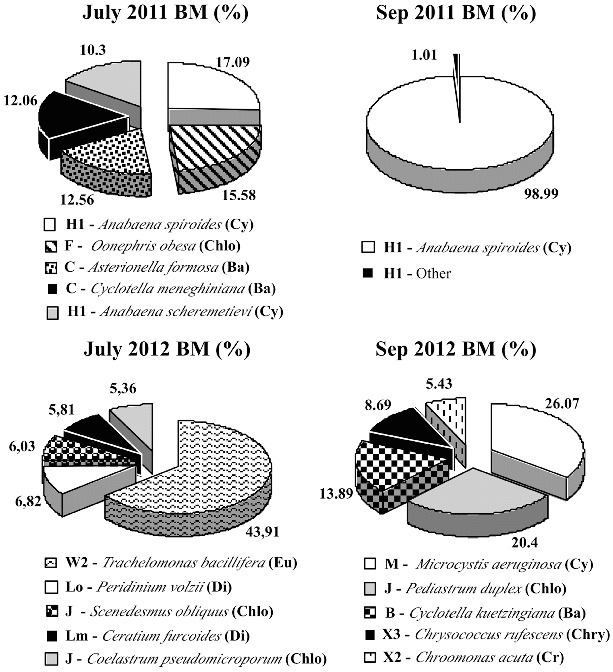
Relative biomass (Bm, %) of the main phytoplankton species (>5% of the total biomass) with their taxonomic and functional groups in Golyamo Skalensko Lake.

The phytoplankton in Golyamo Slakensko Lake differed considerably in the main phytoplankton species (Figure 4) and included five groups that were not found in Malko Skalensko Lake: **H1**, **L_M_, М**, **B** and **W2**. Assemblages **H1**, **L_M_**, **M** are typical for eutrophic habitats. In July 2011, eutrophic blue–green algae from functional group **H1** dominated in biomass (*Anabaena spiroides* – 17.09%, *Anabaena scheremetievi* – 10.3%). This eutrophic assemblage comprises dinitrogen-fixing nostocaleans, which are tolerant to low nitrogen conditions.[29,32] Our results confirmed that the nitrogen compounds in Golyamo Skalensko Lake were on the limit of the analytical method (Table 1). Another dominant group were diatoms (**C**): *Asterionella formosa* (12.56%) and *Cyclotella meneghiniana* (12.06%). The colonial chlorococcalen alga *Oonephris obesa* (**F**) was also represented (15.58%). Other species with negligible biomass were from functional group **L_M_**: *Ceratium furcoides* (0.4%), **Y**: *Cryptomonas marsonii* (0.3%) and **X2**: *Carteria multifilis* (0.1%). Probably, due to increased nitrogen deficiency in the middle of September, *A. spiroides* (**H1**) reached bloom concentrations ( third degree, 65.3 mg L^−1^) and became monodominant with 98.99% of the total phytoplankton biomass (Figure 4). According to Padisák et al.,[35] monodominant blooms are often an indication for hypertrophic conditions.

In 2012, the dominant functional groups in Golyamo Slakensko Lake were completely different from those in 2011. Most of the established functional groups were also typical for Malko Skalensko Lake (**Lo**, **J**, **F**, **C**, **X2** and **X3**), which indicates an improvement in the ecological status. The eutrophic assemblage **H1** was not dominant, but there still remained representatives of two other eutrophic groups in the phytoplankton (**L_M_** and **M**) as main species. In the middle of the summer, the euglenoid alga *Trachelomonas bacillifera* (**W2**) formed almost half of the total biomass; followed by functional group **J**: chlorococcal green algae *Scenedesmus obliquus* (6.03%) and *Coelastrum pseudomicroporum* (5.36%); **Lo** (*Peridinium volzii* – 6.82%); and **L_M_** (*Ceratium furcoides* – 5.81%). In September, the eutrophic functional groups **M** (*Microcystis aeruginosa* – 26.07%) and **J** (*Pediastrum duplex* – 20.4%) dominated. Functional group **B** (*Cyclotella kuetzingiana* – 13.89%), **X3** (*Chrysococcus rufescens* – 8.69%) and **X2** (*Chroomonas acuta* – 5.43%) were also observed.

Thus, the eutrophic state of Golyamo Skalensko Lake in 2011 remains unresolved, given the absence of significant anthropogenic intervention (occasional fish stocking and recreational fishing are the only human activities recorded). It is well known that the main reason for algal blooms is increased levels of total nitrogen and phosphorus [36,37,38] as a result of anthropogenic pressure. It could, therefore, be speculated that there was a sudden release of gases (hydrogen sulphide, etc.) from the cryptofault on which the lake is located. In this region of Stara Planina there have been found several similar faults, including the Skalenski cryptofault.[39] So far, gas releases have been confirmed only in a small lake (Lokvata) located 3 km east of Golyamo Skalensko Lake. Morphometric data for the last 100 years confirm the rapid succession and gradual reduction of its area.[40]

#### **Presence of cyanotoxins**

Taking into account the bloom of the blue–green alga *Anabaena spiroides* (third degree) observed in 2011, collected water samples were analysed for presence of cyanotoxins by HPLC and ELISA. The results were used for toxicological assessment of the state of both investigated lakes.

**Figure 5.**
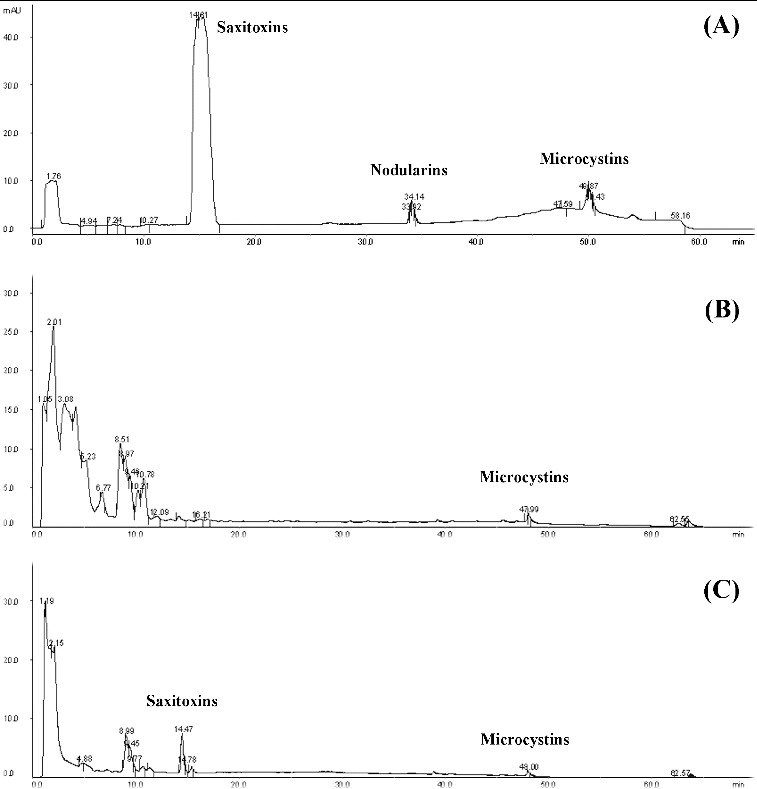
HPLC chromatogram. Standards of cyanotoxins (A); water sample collected in July 2011 from Golyamo Skalensko Lake (B); water sample collected in September 2011 from Golyamo Skalensko Lake (C).

The HPLC analysis (Figure 5) revealed microcystins (hepatotoxins) in the water samples collected from Golyamo Skalensko Lake in July 2011 (peak at 47.99 min) and September 2011 (peak at 48 min). Nodularins were not detected in any of the samples but there were saxitoxins (peak at 14.47 min) in the sample collected in September 2011 (Figure 5(C)). Taking into account the cyanoprokaryotic species identified in these periods, the producers of the detected microcystins were most likely *Anabaena spiroides* (65.33 mg L^−l^ biomass), *Aphanizomenon flos-aquae* (0.01 mg L^−l^ biomass), *Microcystis aeruginosa* (0.05 mg L^−l^ biomass) and *M. wesenbergii* (0.03 mg L^−l^ biomass), which have been repeatedly proven as producers of cyanotoxins.

In the water samples from Golyamo Skalensko Lake collected in 2012 no cyanotoxins were detected. The quantitative development of blue–green algae in 2012 was insufficient (Figure 2(D)). Their relative abundance was from 0.8% to 33.1%, but with lower absolute values of 0.007 mg L^−l^ (July) and 0.43 mg L^−l^ (September), respectively.

As expected, for the entire studied period cyanotoxins were not detected in Malko Skalensko Lake. In the species composition of the phytoplankton, the potential producers of cyanotoxins (*Microcystis aeruginosa* and *Aphanizomenon* flos-aquae) were found only as single specimens in the qualitative samples.

**Figure 6.**
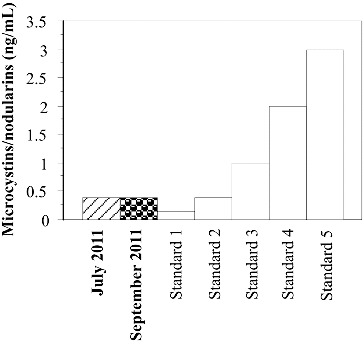
Microcystins/nodularins (ng mL^−1^) in the water samples from Golyamo Skalensko Lake collected in 2011 detected by ELISA.

The ELISA results confirmed the presence of microcystins/nodularins in the water samples from Golyamo Skalensko Lake collected in July and September 2011 (Figure 6). The detected cyanotoxins were at the level of the second standard (0.4 ng mL^−l^). In the water samples from Malko Skalensko Lake cyanotoxins were not detected either in 2011, or in 2012.

#### **Macrophytes**

A total of 14 macrophyte species were recorded. Malko Skalensko Lake supported richer macrophyte diversity (11 taxa, Table 2) with *Potamogeton natans* and *Myriophyllum spicatum* as dominant and deepest species. The shallowest depth zone (0 m to 1 m) supported the highest diversity. Tall reeds such as *Phragmites australis*, *Typha angustifolia*, *T. latifolia* and *Scirpus lacustris* were the most frequent emergent species (helophytes) on the shores. Three woody species were observed: willows (*Salix fragilis* L., *S. triandra* L.) and poplar (*Populus tremula* L.). Six species were registered in Golyamo Skalensko Lake. The species-poor macrophyte community was dominated by *Elodea* species.

**Table 2.** Macrophyte species and vegetation limits, 2011–2012.

	Lake	
	Golyamo Skalensko Lake	Malko Skalensko Lake
Species	*Alisma lanceolatum* With.	*Ceratophyllum demersum* L.
	*Elodea nuttallii* (Planch.) H.St.John	*Elodea canadensis* Michx.
	*Elodea canadensis* Michx.	*Iris pseudacorus* L.
	*Myriophyllum spicatum* L.	*Juncus effusus* L.
	*Polygonum lapathifolium* L.	*Myriophyllum spicatum* L.
	*Typha angustifolia* L.	*Phragmites australis* (Cav.) Trin. ex Steud.
		*Potamogeton lucens* L.
		*Potamogeton natans* L.
		*Scirpus lacustris* L.
		*Typha angustifolia* L.
		*Typha latifolia* L.
Vegetation limit (m)	1.1	3.75

#### **Assessment of the ecological status based on phytoplankton**

The Factor number (*F*), which is needed for calculating the *Q* index, was determined based on the reports of Padisák et al.,[29] Becker et al.,[41,42] Crossetti and Bicudo [43] and expert knowledge. The specific values of *F* for all functional groups that were found in both lakes are listed in Table 3.

**Table 3.** Factor number (*F*) to the phytoplankton functional groups.

Functional group	*F*	Functional group	*F*
**B**	5	**Y**	3
**C**	2.5	**F**	3
**D**	2	**G**	3
**N**	5	J	3
**P**	3	**K**	5
**MP**	5	**H1**	0
**T**	5	**Lo**	5
**S1**	0	**L_M_**	0
**S2**	0	**M**	0
**X3**	5	**W1**	3
**X2**	4	**W2**	4
**X1**	2.5	**W_S_**	4
**E**	5		

The assessment of the ecological status (Table 4) of the two lakes showed that, based on the phytoplankton, Malko Skalensko Lake was on the border of the reference conditions in 2011 and 2012. In July 2011, it was within the reference status for all metrics, except the AGI and transparency, which indicated good status. In September 2011, all indicators indicate again high status, except the Assemblage index (good) and transparency (moderate). In July 2012, the ecological status was slightly deteriorated due to the bloom-forming *Peridinium lomnickii* (first degree), leading to poorer evaluation on a number of metrics (biomass, transparency, chlorophyll-*a*, degree of bloom). Most major indicators showed good ecological status. In September 2012, Malko Skalensko Lake recovered the high status on all metrics, except transparency (good status).

**Table 4.** Ecological assessment based on phytoplankton and macrophytes.

Lake	Malko Skalensko Lake				Golyamo Skalensko Lake			
Year	2011		2012		2011		2012	
Month	July	Sept	July	Sept	July	Sept	July	Sept
Phytoplankton metrics:								
Biomass [BM, mg^3^ l^−l^]	1.35	1.37	**2.95**	1.41	**1.99**	65.99	0.9	1.29
*Q* (Assemblage index)	4.7	**3.6**	4.5	4.2	2.1	0.1	**3.7**	2.7
AGI (Algae Group Index)	**1.01**	0.88	0.31	0.44	3.37	118.7	0.91	**2.39**
Transperancy [SD, m]	**2.05**	1.42	**2.30**	**2.50**	**2.15**	0.42	**2.30**	**2.10**
Chlorophyll-*a* [μg l^−l^]	2.77	2.89	**6.13**	3.22	**4.24**	141.9	1.94	3.31
% Cyanobacteria	3.10	2.04	3.6	0	**30.77**	98.99	0.4	**26.28**
Bloom degree	no	no	**Di-I degree**	no	no	**Cy-III degree**	no	no
Bts (Bloom toxic species)	no Single: Ma	no Single: Ma, Afa	no Single: Ma	no Single: Ma	**Ma, Mw, Afa**	**As-III degree**	**Ma, Mw**	**Ma, Mw**
Ecological status	high	**good**	**good**	high	mod	bad	high	**good**
Macrophytes:								
EQR	**0.7**		**0.7**		**0.07**		**0.07**	

Note. Cy: Cyanobacteria; Di: Dinophyceea; Ma: *Microcystis aeruginosa*; Mw: *Microcystis wesenbergii*; Afa: *Aphanizomenon flos*-*aquae*; As: *Anabaena spiroides*. White with normal font: high ecological status; white with bold font: good ecological status; light grey: moderate (medium) ecological status; dark grey with bold font: poor ecological status; black: bad ecological status.

The assessments for Golyamo Skalensko Lake varied to a high degree during the studied months and years (Table 4), which confirms the well-known fact that phytoplankton is the most dynamic component of the aquatic ecosystems. In 2011, due to the high percentage of Cyanobacteria (July) and the subsequent intense bloom of *Anabaena spiroides* (September), the assessment based on phytoplankton varied between moderate and bad. In 2012, the phytoplankton assessment was dramatically improved and the ecological status of the lake ranged between high (in July) and moderate (in September). This change could be explained by a decrease in the nutrient levels in the lake. Despite the low values of biomass (respectively chlorophyll-*a*) and the lack of blooms, the metrics that reflect the taxonomic composition of the phytoplankton (*Q*, AGI and percentage of Cyanobacteria) indicate worse ecological status: good to poor (Table 4). This is natural, since changes in phytoplankton nutrient levels are usually reflected first in changes in biomass and then in changes in species composition.[3]

In the present study, we tested the feasibility of the Assemblage index for ecological assessment of semi-mountain natural lakes. The index is suitable for monitoring of a variety of ecosystems, as it is based on the concept of habitats (10, 31). According to the authors of the index, the values of the Factor number (*F*) are most critical for its application.[29] They have to be determined for the functional groups of any particular lake type. Due to the lack of paleolimnological data for the phytoplankton of the two lakes, the *F* values determined by us (Table 3) are based primarily on a comparison between the dominant functional groups in both Skalenski Lakes in 2011 (Figures 3 and 4). The phytoplankton status in these lakes showed two extreme scenarios (Table 4: Ecological status, 2011). We gave a maximum value (*F* = 5) for functional groups **N**, **T**, **X3**, **E** and **Lo**, which correspond to unaffected conditions. These groups include the dominant species found in Malko Skalensko Lake in 2011. Lower values (*F* = 3 or 4) were given to **W1** (euglenoids) and **WS** (*Synura*), since they indicate loading of the water with organic matter of vegetable origin. We assigned the lowest value (*F* = 0) to functional groups **H1**, **L_M_** and **M**, since they were found only in Golyamo Skalensko Lake. Intermediate values (*F* = 2.5 and 3) were given to functional groups **C** and **F**, as these groups had dominant representatives in both lakes.

The *Q* and AGI indices give a similar assessment of the ecological status and fit in half of the cases (Table 4). In the other cases, a probable reason for the difference in the assessments is the fact that the formula for calculating AGI does not include some groups, such as Zygnemaphyceae and Euglenophyceae, which in some months had higher relative biomass. As compared to biomass assessment, the indices generally provide a lower ecological assessment (Table 4). This result is in agreement with the finding of Padisák et al. [29] that the ecological status determined by the *Q* index is one class lower than that based on the classical biomass evaluation, because it is sensitive to the occurrence of new species.

#### **Assessment of the ecological status based on macrophytes**

Based on the Reference Index, the status of Malko Skalensko Lake was good, whereas that of Golyamo Skalensko Lake was poor (Table 4). There were degradation indicators and invasive species (*Elodea canadensis*) in both lakes. The monodominant macrophyte community at Golyamo Skalensko Lake indicated an eutrophication process.

Deep macrophyte stands of dominant perennial *Potamogeton natans*, *P. Lucens* and *Myriophyllum spicatum* in Malko Skalensko Lake illustrated good status. Nevertheless, development of the last two insensitive species could be related to an elevated level of primary production. *M. spicatum* is relatively tolerant to pollution, salinity and pH values, and was found to be positively correlated with segmentation of lakes.[44]

#### **Type-specific reference species**

The proposed list of general type-specific reference species (Table 5) for national L4 type does not reflect the actual species composition for all lakes in a high condition within the type. Instead, it provides an open “pool of species” with room for further additions in the course of increasing knowledge and number of studied lakes.

**Table 5.** Alphabetic list of preliminary type-specific taxa in semi-mountain natural lakes.

Phytoplankton – taxa	Macrophytes – taxa
Cyanobacteria	Truly aquatic plants (hydrophytes)
*Chroococcus limneticus* Lemmermann	*Myriophyllum spicatum* L.
*Snowella arachnoidea* Komárek et Hindák	*Potamogeton lucens* L.
*Woronichinia naegeliana* (Unger) Elenkin	*Potamogeton natans* L.
Chlorophyceae (Green algae)	Emergent species (helophytes)
*Crucigenia tetrapedia* (Kirchner) West et G.S. West	*Iris pseudacorus* L.
*Kirchneriella lunaris* (Kirchn.) Moeb.	*Juncus effusus* L.
*Oocystis marssonii* Lemm.	*Phragmites australis* (Cav.) Trin. ex Steud.
*Radiofilum conjunctivum* Schmidle	*Scirpus lacustris* L.
*Tetraedron caudatum* (Corda) Hansg.	*Typha angustifolia* L.
Zygnemaphyceae	*Typha latifolia* L.
*Cosmarium depressum* (Nägeli) P. Lundell	
*Cosmarium moniliforme* Turpin ex Ralfs	
*Sphaerozosma vertebratum* (Bréb.) Ralfs	
*Zygnema* sp.	
Chrysophyceae	
*Chrysococcus cordiformis* Naumann	
*Chrysococcus rufescens* Klebs	
*Dinobryon crenulatum* W. et G.S. West	
*Kephyrion litorale* J.W.C. Lumd	
*Kephyrion rubri-claustri* Conr.	
*Mallomonas caudata* Iwanoff	
Dinophyceae	
*Ceratium furcoides* (Levander) Langhans	
*Ceratium hirundinella* (O.F.Müller) Dujardin	
*Peridinium aciculiferum* Lemmermann	
*Peridinium cinctum* (O.F.M.) Ehr.	
Cryptophyceae	
*Chroomonas acuta* Uterm.	
*Chroomonas coerulea* (Geitler) Skuja	
Euglenophyceae	
*Euglena proxima* Dang.	
*Phacus monilatus* Stokes var. *suecicus* Lemm. (Eichwald) Lemmermann	

## Conclusions

Both Skalenski Lakes are not subjected to anthropogenic pressure, but have a different assessment of the ecological status. According to the phytoplankton assessment, Malko Skalensko Lake is on the border of the reference conditions, but in good status according to the macrophytes. Golyamo Skalensko Lake was assessed to be in poor status based on macrophytes. Phytoplankton metrics indicated moderate and bad status in 2011, and high and moderate status in 2012. Cyanobacterial blooms during the first year of observation cannot be related to anthropogenic pressure, but could rather reflect the influence of natural gases (hydrogen sulphide, etc.) coming from gas conductive faults of the East Stara Planina Mountain. Phytolpankton-based assessment showed temporary ecosystem alterations and different assessment results during the period 2011–2012 in response to nutrient levels. In addition, the macrophytes illustrated an eutrophication process, which should be monitored and managed in order to improve the lake potential. Toxicological assessment by HPLC and ELISA showed presence of hepatotoxins in Golyamo Skalensko Lake in 2011. Although the detected concentrations were lower than the limit levels (1 μg L^−1^), Cyanobacteria and the toxins produced by them have to be monitored in the lakes.

Two BQEs and eight phytoplankton metrics were applied, instead of only one (e.g. chlorophyll-*a*), to obtain a more complete picture of the pressures: a fast response of the phytoplanktonic biomass and composition illustrates the momentary status, while macrophytes provide information on long-term environmental alterations. Choosing the appropriate assessment metrics and providing standardized classification systems in compliance with the requirements of WFD depends on the specific and unique lake conditions. In general, establishment of natural variability of the lake reference conditions is necessary. This will provide important information for the lake management and successful restoration if needed. 
